# Repeatability and agreement of white-to-white measurements between slit-scanning tomography, infrared biometry, dual rotating Scheimpflug camera/Placido disc tomography, and swept source anterior segment optical coherence tomography

**DOI:** 10.1371/journal.pone.0254832

**Published:** 2021-07-16

**Authors:** Alexander Buckenham Boyle, Soobin Namkung, William Shew, Akilesh Gokul, Charles N. J. McGhee, Mohammed Ziaei

**Affiliations:** 1 Faculty of Medical and Health Sciences, Department of Ophthalmology, New Zealand National Eye Centre, The University of Auckland, Auckland, New Zealand; 2 Department of Ophthalmology, Greenlane Clinical Centre, Epsom, Auckland, New Zealand; University of Toronto, CANADA

## Abstract

**Purpose:**

To assess the agreement and repeatability of horizontal visible iris diameter (HVID) or white-to-white (WTW) measurements between four imaging modalities; combination slit scanning elevation/Placido tomography, infrared biometry, dual rotating scheimpflug camera/Placido tomography, and swept source anterior segment optical coherence tomography (AS-OCT).

**Methods:**

A prospective study of 35 right eyes of healthy volunteers were evaluated using the Orbscan IIz, IOL Master 700, Galilei G2, and DRI Triton OCT devices. The inter-device agreement and repeatability of HVID/WTW measurements for each device were analysed.

**Results:**

Mean HVID/WTW values obtained by the Orbscan IIz, IOL Master 700, Galilei G2 and DRI Triton OCT were 11.77 ± 0.40 mm, 12.40 ± 0.43 mm, 12.25 ± 0.42 mm, and 12.42 ± 0.47 mm, respectively. All pairwise comparisons revealed statistically significant differences in mean HVID/WTW measurements (p = <0.01) except for the IOL Master 700—DRI OCT Triton pair (p = 0.56). Mean differences showed that the DRI Triton OCT produced the highest HVID/WTW values, followed by the IOL Master 700, Galilei G2 and Orbscan IIz, respectively. The limits of agreement were large on all device pairs. There was high repeatability for all devices (ICC ≥ 0.980). The highest repeatability was seen in the Galilei G2 (ICC = 0.995) and lowest in the Orbscan IIz (ICC = 0.980).

**Conclusions:**

The four devices exhibit high repeatability, but should not be used interchangeably for HVID/WTW measurements in clinical practice.

## Introduction

The horizontal visible iris diameter (HVID) or white-to-white (WTW) measurement is defined as the horizontal visible extent of the iris. The HVID/WTW measurement is used in contact lens fitting, intraocular lens power calculation, diagnosis of various corneal diseases (e.g. megalocornea) and in the monitoring of buphthalmos in childhood glaucoma [1].

Accurate estimation of horizontal internal anterior chamber diameter (HIACD) is also crucial when sizing angle supported anterior chamber intraocular lenses (ACIOL), as well as phakic intraocular lenses (pIOL) such as the implantable collamer lens (ICL). Accurate sizing of such implants can prevent postoperative complications such as endothelial cell loss, cataract formation, chronic inflammation and raised intraocular pressure [2].

For contact lens fitting, the HVID generates the total diameter, optic zone diameter and the peripheral curves necessary to ensure there is adequate limbal clearance with an even distribution of scleral pressure in large diameter designs [3]. The HVID is also an important component of the sag calculation necessary to optimise the corneal clearance. Therefore repeatable HVID measurements are of significant importance for contact lens fitting.

There are several techniques commonly used to measure the HVID/WTW distance. These can be divided into manual techniques, for instance, surgical calipers, corneal gauges, or scales in slit-lamp ocular rings, and automated techniques, such as ultrasonic biomicroscopy, anterior segment optical coherence tomography (AS-OCT), direct imaging techniques, or magnetic resonance imaging. Automated measurements have previously been shown to provide more precise results than manual techniques [4].

Given the wide variety of techniques used to measure HVID/WTW distance, the clinical significance of HVID/WTW measurement repeatability and inter-device agreement, and the use of HVID/WTW as a proxy of horizontal internal anterior chamber diameter (HIACD), the current study sought to investigate repeatability and inter-device agreement of HVID/WTW measurements using the Orbscan IIz (slit-scanning corneal tomography), IOL Master 700 (infrared biometry), Galilei G2 (dual rotating scheimpflug camera/Placido tomography) and DRI OCT Triton (swept source anterior segment optical coherence tomography).

## Material and methods

### Enrolment

This study enrolled 35 healthy participants aged 18 to 25. Participants were all students attending The University of Auckland Medical School and were recruited over a period of one week. Exclusion criteria included a history of corneal abnormalities, active ocular pathology, previous ocular surgery, media opacity, poor corrected visual acuity and fixation instability. The study was approved by the University of Auckland Human Participants Ethics Committee (approval number 022624). Informed consent was obtained from all participants after they voiced understanding of the purpose and the procedures of the study in accordance with the tenets of the Declaration of Helsinki.

### Acquisition

Participants were scanned using the Orbscan IIz (Bausch & Lomb, USA, version 3.12), IOL Master 700 (Carl Zeiss Meditec AG, Germany, version 1.7), Galilei G2 (Zeimer Ophthalmic Systems, Switzerland, version 6.1.3,) and the DRI OCT Triton (Topcon, USA, version 10.13) in a random manner. All four devices were calibrated as per manufacturer recommendations prior to beginning data collection.

Only the right eye of each participant was selected and subsequently scanned three times thereby obtaining three good quality acquisitions by each device for each subject. All measurements were conducted in a darkened room without the use of any eye drops. With their chins on the chinrest, participants were asked to fixate on the target light and to blink completely immediately prior to each measurement to allow for adequate tear-film coverage over the corneal surface. They were then asked to open their eyes and hold for the duration of the scan to minimise interference of the eyelids at the limbus.

The investigators checked the quality of each scan immediately after acquisition and only scans of acceptable quality were included. The means of determining ‘acceptable quality’ was dependent on the device used and the criteria provided by each device’s manufacturer. The Orbscan IIz automatically and immediately discards measurements deemed to be of unacceptable quality. The Galilei G2 provides an overall quality score taking into account Scheimpflug/Placido images, motion compensation, and motion distance, and scans were repeated until three with acceptable overall quality scores were obtained [5]. Likewise, the IOL Master 700 provides an automated quality check for each component of the scan e.g. alignment. Scans were repeated on the IOL Master 700 until three scans were obtained with acceptable quality for all components of the scan. Anterior segment OCT images were reviewed by the same experienced examiner, and those not capturing the widest HVID/WTW point or of poor quality were repeated until three subjectively acceptable scans were obtained. HIACD was then measured manually using a digitally calibrated caliper between the vertexes of the two iridocorneal angles at the horizontal meridian as previously described by Kohnen et al. [6]. In addition to the above in-built quality controls, topography images were inspected by the investigator to detect erroneous measurements e.g. as result of lid interference, not detected by the device. The HVID/WTW values for three consecutive high-quality scans were recorded for each device.

### Devices

All four devices determine HVID/WTW through different means. The Orbscan IIz uses both Placido rings and slit scanning for topography measurements. One hundred and forty slit grey scale images are captured in conjunction with the Placido disc image to automatically calculate the HVID/WTW distance [7].

The IOL Master 700 captures the HVID/WTW from an infrared digital grey-scale photograph of the anterior segment taken after focusing on the iris via automated detection of the limbus, similar to the Orbscan IIz [4].

The Galilei G2 uses dual-scheimpflug camera tomography to visualise both the anterior and posterior corneal surfaces. For HVID/WTW measurement the limbus is automatically fitted with a best-fit-ellipse in reference to a top view image provided by a high definition camera. The maximum length in the horizontal direction of the ellipse (not the long or short axis) is taken as the naso-temporal limbus parameter [8].

The DRI OCT Triton device has a scanning wavelength of 1,050nm and with the anterior segment attachment, high-resolution images of the anterior chamber to the periphery of the cornea can be taken [9]. From these images an in-built digital caliper function can be used to measure the HIACD. Although the values obtained using DRI OCT Triton are HIACD measurements, the values are referred to as HVID/WTW measurements throughout this paper for simplicity and ease of reading.

### Statistical analysis

SPSS 19.0 for Windows (SPSS, IBM, Chicago, Illinois, USA) and Microsoft Excel (version 16.20, Microsoft Corporation, Redmond, WA, USA) were used for statistical analysis. The Kolmogorov-Smirnov test for data normality was run and the results indicated that the data were normally distributed. Repeatability for each device was assessed using within-subject standard deviation (Sw), within-subject coefficient of variation (Cfvar), precision, repeatability (intra-session test-retest variability), and the intraclass correlation coefficient (ICC). The Cfvar was expressed as a percentage calculated by dividing Sw by the overall mean. As such, Cfvar is a measure of the variation in measurements relative to the mean, indicative of the relative amount of noise in the system, while Sw is an absolute measure of variation, indicative of the overall variability of measurements. Highly repeatable devices have both a low Sw and Cfvar (<1%). Precision, representing the difference between the true value and a subject’s measurement, was calculated as 1.96 x Sw. Repeatability was calculated as 2.77 x Sw. ICC was calculated as the ratio of the between-subject variance to the sum of the combined within-subject and between-subject variance. A value of 1 would represent perfect agreement, and a value of 0 would represent no agreement.

For inter-device agreement, the method recommended by Bland and Altman, including the Pearson correlation coefficient, was used [10]. The mean and the difference between the measurements for all possible pairwise comparisons for the different devices were calculated and plotted. The Bland-Altman analysis indicates if a fixed and proportional bias exists between device pairs. A fixed bias indicates that the difference in measurements obtained by one device is consistently higher or lower than the other device by a relatively constant amount. A proportional bias indicates that the difference in measurements between a device pair has a linear relationship and the difference in measurements is dependent on the magnitude of the measurement. In addition to this, the following figures were calculated and represented on the plots; mean difference, 95% confidence interval of the average difference, and 95% limits of agreement (LoA). The LoA were calculated as mean difference ± 1.96 standard deviations (SD). A p-value of less than 0.05 was considered statistically significant. Assessment of similar investigations of agreement of HVID/WTW in different devices revealed that the mean difference between device pairs where a significant difference was detected ranged from 0.33mm– 0.51mm with standard deviations ranging from 0.11 mm– 0.78 mm [2,7,11]. A sample calculation to distinguish a mean difference of 0.30 mm with a standard deviation of 0.50 mm at a significance level of 0.05 and power of 80% revealed that minimum sample size of 34 eyes were required for agreement and, a minimum of 32 eyes were required for repeatability to produce a confidence limit of 20% of Sw.

## Results

Thirty-five right eyes (21 female) were assessed and included in the analysis. There were no signs of corneal disease evident on computerised corneal tomography for any participants. The mean age of participants was 22.9 ± 1.57 years.

### Repeatability of HVID/WTW measurements

The repeatability parameters for the HVID/WTW measurements in each device are summarised in Table 1. The Galilei exhibited the highest Sw, Cfvar and ICC while the Orbscan IIz exhibited the lowest Sw, Cfvar and ICC. Overall, repeatability was high with ICC ≥ 0.980 for all devices.

**Table 1:** Intra-observer repeatability of white-to-white/horizontal internal anterior chamber diameter in control subjects using the Orbscan IIz, IOL Master 700, Galilei G2 and DRI OCT Triton Anterior OCT.

Device	Mean ± SD (mm)	Within-Subject SD	Precision	Repeatability	Cfvar (%)	ICC
Orbscan IIz	11.77 ± 0.40 (11.37–12.17)	0.071	0.139	0.197	0.609	0.980
IOL Master 700	12.40 ± 0.43 (11.97–12.82)	0.053	0.104	0.146	0.428	0.990
Galilei G2	12.25 ± 0.42 (11.83–12.67)	0.032	0.063	0.089	0.264	0.995
DRI OCT Triton Anterior OCT	12.42 ± 0.47 (11.95–12.89)	0.056	0.110	0.155	0.452	0.993

SD = standard deviation; Cfvar = coefficient of variation; ICC = intraclass correlation coefficient.

### Agreement of HVID/WTW measurement between devices

Agreement parameters are summarised in Table 2. All pairwise comparisons revealed statistically significant differences in mean HVID/WTW measurement as calculated using a one-sample T-test on SPSS 19.0 (p<0.001) except for the IOL Master 700—DRI OCT Triton pair (p = 0.56).

**Table 2:** Agreement of white-to-white/horizontal internal anterior chamber diameter between the Orbscan IIz, IOL Master 700, Galilei G2 and DRI OCT Triton Anterior OCT.

Agreement	Mean Difference (mm) ± SD	Significance (2 tailed)	Fixed Bias	Proportional Bias	95% LoA
Orbscan IIz-IOL Master 700	-0.628 ± 0.147	<0.001	Yes	No	-0.917 to -0.339
Orbscan IIz-Galilei G2	-0.481 ± 0.110	<0.001	Yes	No	-0.695 to -0.266
Orbscan IIz- DRI OCT Triton Anterior OCT	-0.652 ± 0.187	<0.001	Yes	No	-1.018 to -0.286
IOL Master 700 –Galilei G2	0.147 ± 0.108	<0.001	Yes	No	-0.064 to 0.358
IOL Master 700- DRI OCT Triton Anterior OCT	-0.024 ± 0.247	0.562	No	No	-0.508 to 0.459
Galilei G2 –DRI OCT Triton Anterior OCT	-0.171 ± 0.191	<0.001	Yes	No	-0.545 to 0.202

SD = standard deviation.

**Fig 1:**
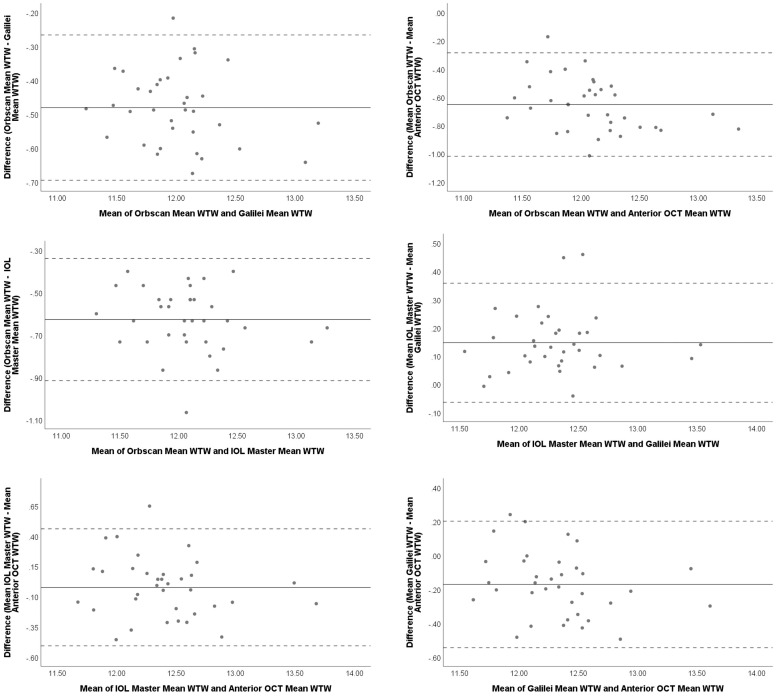
Bland-Altman plots for horizontal visible iris diameter (HVID)/white-to-white(WTW) measurements between device pairs (mm). Solid lines show the mean of the difference between the 2 devices while dotted lines represent 95% limits of agreement.

Bland-Altman plots for agreement of HVID/WTW measurements are shown in Fig 1. The 95% LoA was smallest between IOL Master 700 and Galilei G2 and largest between IOL Master 700 and DRI OCT Triton Anterior OCT.

## Discussion

This study examined the inter-device agreement and repeatability of HVID/WTW measurements using 4 widely used instruments, the Orbscan IIz, IOL Master 700, Galilei G2, and DRI Triton OCT. All devices exhibited high repeatability, but all pairwise comparisons revealed statistically significant differences in mean HVID/WTW measurement except for the IOL Master 700—DRI OCT Triton pair.

WTW measurements have widespread applications in ophthalmology and optometry. Most modern IOL formulae, including the Barrett Universal II formula, which has been shown to achieve superior refractive outcomes post-operatively [12–14], utilise WTW measurements in their calculations. WTW measurements are also crucial for accurate sizing of implantable contact lenses (ICL) as it serves as a surrogate for sulcus diameter. Underestimation may result in a low vault and early cataract formation, whereas overestimation may drive an increase in intraocular pressure and cause pupillary block and secondary glaucoma. The food and drug administration (FDA) study reported that explantation of ICLs due to inaccurate sizing is a rare phenomenon (<0.8%) [15], however one study revealed that 70% of explanted ICLs are secondary to inaccurate sizing [16]. Considering that these lenses are sized to the nearest 0.50 mm, it has been suggested that a difference equal to or more than 0.50 mm in the WTW distance is clinically significant [15] Whilst there is no current gold standard method for WTW measurements, the ICL lens sizing protocol originally approved by the United States FDA in 2005 requires adding 0.5mm to the horizontal white-to-white measurement obtained using calipers at a slit lamp or using the Orbscan unit [15]. This method of determining the ICL size relies on the assumption that there is a correlation between the white-to-white distances and the sulcus iridociliaris [17,18]. This has led to many surgeons relying upon Orbscan derived WTW measurements for ICL surgery as automated methods rather than caliper use have been reported to have reduced inter-operator variability and greater accuracy [4]. As such, when we compare the IOL Master 700, Galilei G2 and DRI OCT Triton to the Orbscan IIz, we note that all three devices produce a larger measurement of WTW/HIACD than the Orbscan IIz with a fixed bias only. The bias was negative with the lower limit of the 95% LoA being as low as -1.018 mm, indicating that most devices may produce a lower HVID/WTW measurement leading to calculations that can produce oversized ICLs. Since the IOL Master 700, Galilei G2 and DRI OCT Triton are all highly repeatable, with the Galilei G2 being most repeatable, all three devices can be used in calculations of ICL size, however, the agreement with Orbscan IIz results indicate that compensation may be required when calculating ICL size pre-operatively.

As well as these surgical implications, WTW measurement is important in the diagnosis and monitoring of various corneal diseases [1]. Over the last decade, increasing interest in scleral lens designs have resurfaced and the most important consideration when designing these lenses is the diameter—all curvatures and power are based around this parameter. Thus the importance of revisiting the forgotten HVID is warranted.

The corneal scleral limbus is histologically complex and the interface of the transition points between sclera and cornea do not align exactly in a superficial to deep fashion. Therefore, several definitions of the histological and clinical limbus have been proposed. The clinical definition continues to rely on identifying the inner limit of the blue-grey ring of the cornea (ie the HVID/WTW) and clinically labelling the limbus as a 1.5 to 2.0mm band of tissue surrounding this.

The indeterminate nature of the corneal blue-grey ring makes the HVID/WTW an inherently subjective measurement. Various studies have reported HVID/WTW measurements in normal individuals using a variety of devices [6,19–21]. Although HVID/WTW distance can be measured manually using calipers [4,20], automated devices which typically detect the corneal limbus by comparing grey-scale steps [22], have been shown to provide a more precise and reliable reading [3].

This study sought to examine the repeatability and agreement of automated HVID/WTW measurements using the Orbscan IIz, IOL Master 700 and Galilei G2, as well as manual HIACD measurements on the DRI OCT Triton device, in healthy eyes.

The results show a high level of repeatability in all four devices, with Galilei G2 demonstrating the highest level of repeatability and Orbscan IIz the lowest. All pairwise comparisons revealed statistically significant differences in mean HVID/WTW measurement except for the IOL Master 700- DRI OCT Triton pair. The DRI OCT Triton measured the largest HVID/WTW values, followed by IOL Master 700, Galilei G2 and Orbscan IIz respectively. On average DRI OCT Triton showed values approximately 0.02mm above the IOL Master 700, 0.17mm above Galilei G2 and 0.65mm above Orbscan IIz. Orbscan IIz measured the lowest values by a significant margin which is in keeping with previous studies [22]. The results reveal a fixed bias between all device pairs except the IOL Master 700- DRI OCT Triton pair, and no proportional bias between any device pairs.

Bland-Altman plots showed wide LoA for all pairwise comparisons. The widest range of LoA was obtained for the comparison between the IOL Master 700 and DRI OCT Triton devices. Although IOL Master 700 and DRI OCT Triton showed the highest level of agreement, the wide LoA of this device pair and the significant variation in measured HVID/WTW distance as seen on the Bland Altman plot indicates that for some eyes the measurements had poor agreement.

These findings, together with the fixed bias apparent between the other pairwise comparisons, suggest that HVID/WTW measurements cannot be used interchangeably between devices. Care should therefore be taken if one device is replaced with another or when multiple devices are used to take measurements. Overall, the differences between these devices are most likely related to the way they each define the limbus, and their different methodologies, optical principles and light sources, namely the brightness of the light source used in each device [23]. In addition to this, the level at which each device determines whether a scan is of acceptable quality may be a contributing factor as scan quality is likely to influence accuracy of measurement. Furthermore, although participants were instructed to blink before each scan, varying acquisition times with each device may have led to various degrees of artefact from tear evaporation, corneal irregularity, or head tilt, all of which may influence HVID/WTW measurement.

The DRI OCT Triton should technically produce a better HIACD measurement than the other devices given it visualises the anterior chamber directly rather than using the cornea/limbus to obtain its measurement. Given the lack of a statistically significant difference in mean HVID/WTW measurement for the IOL Master 700, and mean HIACD measured by DRI OCT Triton, it may be that HVID/WTW measurements obtained using IOL Master 700 correlate with HIACD measurements more closely than HVID/WTW measurements using the Orbscan IIz or Galilei G2. However differences between the IOL Master 700 measurements and the DRI OCT Triton measurements may have been masked by averaging out of extreme values and the small sample size of our study. Kohnen *et al*. compared HVID/WTW measurements using IOL Master and Orbscan IIz with HIACD measurements from Visante OCT (Carl Zeiss Meditec AG, Germany) and reported that HIACD measurements were 0.36mm larger than the mean IOL Master HVID/WTW measurements and 0.68mm larger than those obtained by the Orbscan IIz [6].

All three devices which measured HVID/WTW distance exhibited smaller mean values than the mean HIACD values from DRI Triton OCT. This may reflect the innate subjectivity of anterior OCT derived HVID/WTW measurements, which is investigator dependent in terms of both alignment of the scan at the widest horizontal HVID/WTW distance and then in the actual use of the digital calipers in which the investigator defines the angle. This subjectivity, as well as the learning curve associated with anterior OCT use, mean that no gold standard method for measuring HIACD is currently available.

Dominguez-Vicent *et al*. completed a study examining HVID/WTW interchangeability between five devices. The group reported comparable measurements between IOL Master 500 and Orbscan II. They concluded that Orbscan II and IOL Master 500 can be used interchangeably to measure HIACD [11]. This conflicts with our findings which imply Orbscan IIz and IOL Master 700 should not be used interchangeably. This could be a result of variability between IOL Master 700 and IOL Master 500 as a -0.101 mm difference in HVID/WTW measurement between the two devices has previously been reported [24].

It is important to note that a previous study comparing OA-2000 (an optical biometer and topography-keratometer) and IOL Master 500 in keratometry, axial length, anterior chamber depth, and HVID/WTW diameter, reported strong agreement between the two devices for almost all biometric measurements, except for the HVID/WTW diameter [25]. This could reflect an innate variability in assessing HVID/WTW diameter compared to other measurements.

This study has several limitations. The participants included were young, healthy individuals and it is possible that results may differ in corneal pathology such as scarring, arcus, keratoconus and also elderly patients or patients following intraocular surgery. Other potential limitations include a small sample size and the lack of refractive error assessment of participants, however previous studies have shown WTW measurements are directly correlated with spherical equivalent due to the role of corneal radius of curvature in different types of refractive error [26].

### Conclusion

HVID/WTW measurements cannot be used interchangeably between the studied devices in healthy eyes. There are differences in repeatability of measurements taken in all four devices, with the Orbscan IIz showing the lowest repeatability and the Galilei G2 the highest. The clinical importance of accurate measurement for large diameter contact lens fitting, refractive surgical planning, diagnosis and monitoring of corneal disease/paediatric glaucoma mean that caution should be exercised when using multiple devices concurrently or when replacing one device with another.

## Supporting information

S1 Data WTW data. (XLSX)

## References

[1] Wallace D, Plager D. Corneal Diameter in Childhood Aphakic Glaucoma. *J Pediatr Ophthalmol Strabismus*. 1996;33:230–234. 8880615 10.3928/0191-3913-19960901-06

[2] Kim S, Kim H, Song J. Comparison of Internal Anterior Chamber Diameter Imaging Modalities: 35-MHz Ultrasound Biomicroscopy, Visante Optical Coherence Tomography, and Pentacam. Journal of Refractive Surgery. 2010;26(2):120–126. doi: 10.3928/1081597X-20100121-07 20163076

[3] Vincent SJ, Alonso-Caneiro D, Collins MJ. Optical coherence tomography and scleral contact lenses: clinical and research applications. Clin Exp Optom. 2019 May;102(3):224–241. doi: 10.1111/cxo.12814 30062745

[4] Baumeister M, Terzi E, Ekici Y, Kohnen T. Comparison of manual and automated methods to determine horizontal corneal diameter. Journal of Cataract & Refractive Surgery. 2004;30(2):374–380. doi: 10.1016/j.jcrs.2003.06.004 15030827

[5] Ziaei M, Meyer J, Gokul A, Vellara H, McGhee CNJ. Direct measurement of anterior corneal curvature changes attributable to epithelial removal in keratoconus. J Cataract Refract Surg. 2018 Jan;44(1):71–77. doi: 10.1016/j.jcrs.2017.10.044 29502621

[6] Kohnen T, Thomala M, Cichocki M, Strenger A. Internal anterior chamber diameter using optical coherence tomography compared with white-to-white distances using automated measurements. Journal of Cataract & Refractive Surgery. 2006;32(11):1809–1813. doi: 10.1016/j.jcrs.2006.08.023 17081862

[7] Salouti R, Nowroozzadeh M, Zamani M, Ghoreyshi M, Khodaman A. Comparison of Horizontal Corneal Diameter Measurements Using the Orbscan IIz and Pentacam HR Systems. Cornea. 2013;32(11):1460–1464. doi: 10.1097/ICO.0b013e3182a40786 24055904

[8] Salouti R, Nowroozzadeh M, Zamani M, Ghoreyshi M, Salouti R. Comparison of horizontal corneal diameter measurements using Galilei, EyeSys and Orbscan II systems. Clinical and Experimental Optometry. 2009;92(5):429–433. doi: 10.1111/j.1444-0938.2009.00407.x 19681922

[9] DRI OCT Triton swept source OCT [Internet]. Topcon-medical.eu. 2020 [cited 10 April 2020]. https://www.topcon-medical.eu/eu/products/382-dri-oct-triton-swept-source-oct.html#description.

[10] Bland J, Altman D. Statistical Methods for Assessing Agreement Between Two Methods of Clinical Measurement. The Lancet. 1986;327(8476):307–310. 2868172

[11] Domínguez-Vicent A, Pérez-Vives C, Ferrer-Blasco T, Albarrán-Diego C, Montés-Micó R. Interchangeability among five devices that measure anterior eye distances. Clinical and Experimental Optometry. 2015;98(3):254–262. doi: 10.1111/cxo.12247 25786342

[12] Kohnen T. Emmetropization in cataract surgery. Masket S, Crandall AS, eds, Atlas of Cataract Surgery London: Martin Dunitz, 1999 p 159–167 10.

[13] Roberts T, Hodge C, Sutton G, Lawless M. Comparison of Hill-radial basis function, Barrett Universal and current third generation formulas for the calculation of intraocular lens power during cataract surgery. Clinical & Experimental Ophthalmology. 2017;46(3):240–246. doi: 10.1111/ceo.13034 28778114

[14] Wang Q, Jiang W, Lin T, Zhu Y, Chen C, Lin H et al. Accuracy of intraocular lens power calculation formulas in long eyes: a systematic review and meta-analysis. Clinical & Experimental Ophthalmology. 2018;46(7):738–749. doi: 10.1111/ceo.13184 29498180

[15] Sanders DR, Vukich JA, Doney K, Gaston M, Implantable Contact Lens in Treatment of Myopia Study G. U.S. Food and Drug Administration clinical trial of the Implantable Contact Lens for moderate to high myopia. Ophthalmology 2003; 110: 255–266. 12578765 10.1016/s0161-6420(02)01771-2

[16] AlSabaani NA, Behrens A, Jastanieah S, Al Malki S, Al Jindan M, Al Motowa S. Causes of Phakic Implantable Collamer Lens Explantation/Exchange at King Khaled Eye Specialist Hospital. Middle East Afr J Ophthalmol 2016; 23: 293–295. doi: 10.4103/0974-9233.194076 27994391 PMC5141621

[17] Reinstein DZ, Archer TJ, Silverman RH, Rondeau MJ, Coleman DJ. Correlation of anterior chamber angle and ciliary sulcus diameters with white-to-white corneal diameter in high myopes using Artemis VHF digital ultrasound. J Refract Surg 2009;25:185–194.3. doi: 10.3928/1081597X-20090201-03 19241769 PMC2649749

[18] Gao J, Liao R-F, Li N. Ciliary sulcus diameters at different anterior chamber depths in highly myopic eyes. J Cataract Refract Surg 2013;39:1011–1016. doi: 10.1016/j.jcrs.2013.01.040 23582363

[19] Werner L, Izak A, Pandey S, Apple D, Trivedi R, Schmidbauer J. Correlation between different measurements within the eye relative to phakic intraocular lens implantation. Journal of Cataract & Refractive Surgery. 2004;30(9):1982–1988. doi: 10.1016/j.jcrs.2003.10.041 15342066

[20] Pop M, Payette Y, Mansour M. Predicting sulcus size using ocular measurements. Journal of Cataract & Refractive Surgery. 2001;27(7):1033–1038. doi: 10.1016/s0886-3350(00)00830-0 11489572

[21] Goldsmith J, Li Y, Chalita M, Westphal V, Patil C, Rollins A et al. Anterior chamber width measurement by high-speed optical coherence tomography. Ophthalmology. 2005;112(2):238–244. doi: 10.1016/j.ophtha.2004.09.019 15691557 PMC1784115

[22] Piñero D, Plaza Puche A, Alió J. Corneal diameter measurements by corneal topography and angle-to-angle measurements by optical coherence tomography: Evaluation of equivalence. Journal of Cataract & Refractive Surgery. 2008;34(1):126–131. doi: 10.1016/j.jcrs.2007.10.010 18165092

[23] Bjelos RM, Busic M, Cima I, Kuzmanovic EB, Bosnar D, Miletic D. Intraobserver and interobserver repeatability of ocular components measurement in cataract eyes using a new optical low coherence reflectometer. Graefes Arch Clin Exp Ophthalmol 2011; 249: 83–87. doi: 10.1007/s00417-010-1546-z 20981435

[24] Srivannaboon S, Chirapapaisan C, Chonpimai P, Loket S. Clinical comparison of a new swept-source optical coherence tomography–based optical biometer and a time-domain optical coherence tomography–based optical biometer. Journal of Cataract & Refractive Surgery. 2015;41(10):2224–2232. doi: 10.1016/j.jcrs.2015.03.019 26703299

[25] Kongsap P. Comparison of a new optical biometer and a standard biometer in cataract patients. Eye and Vision. 2016;3(1). doi: 10.1186/s40662-016-0059-1 27833928 PMC5066293

[26] Hashemi H, Khabazkhoob M, Emamian MH, Shariati M, Yekta A, Fotouhi A. White-to-white corneal diameter distribution in an adult population. J Curr Ophthalmol. 2015 Oct 19;27(1–2):21–4. doi: 10.1016/j.joco.2015.09.001 27239570 PMC4877715

